# Supplementary material for: Impacts of Captive Domestication and Geographical Divergence on the Gut Microbiome of Endangered Forest Musk Deer
Source: Animals (Basel). 2025 Jul 2;15(13):1954. doi: 10.3390/ani15131954 (PMC12248866; doi:10.3390/ani15131954)
Supplement: Supplementary file 1 [file animals-15-01954-s001.zip › animals-3710845-supplementary.pdf]

animals

MDPI

Supplementary Materials

1. Supplementary Figures

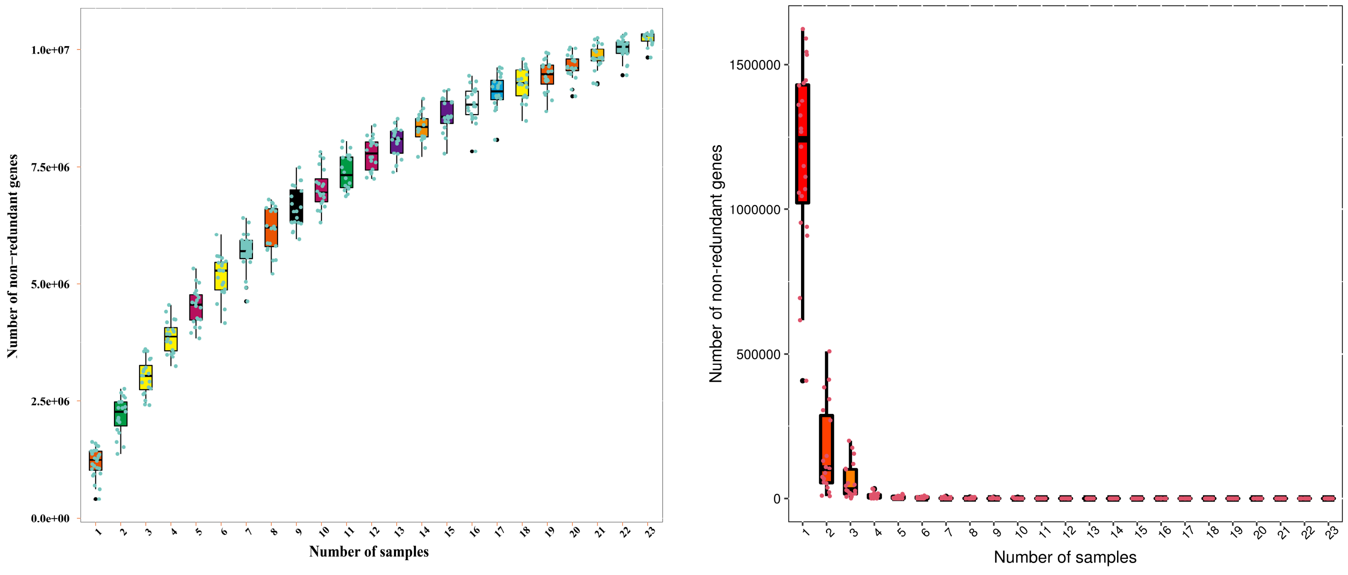

(a) (b)

Figure S1. Core-pan gene analysis. Starting from the gene abundance matrix across samples, the gene count information for each sample was obtained. By randomly sampling different numbers of specimens, the gene counts across varying combinations of sampled specimens were calculated, thereby constructing and plotting the rarefaction curves for Core and Pan genes. (a) Pan gene rarefaction curve; (b) Core gene rarefaction curve.

2. Supplementary Tables

Table S1. Sample Information Table (CQ1-CQ8: W1 group; HN1-HN8: W2 group; C1-C8: C group)

Sample ID	**Time**	**Collection Location**	**Geo-graphic Co-**	**Eleva-tion**	**Vegetation Type**	**Primary Food Source(s)**
CQ1	2023/7/9	Jinfo Mountain	107°03′–107°26′E, 28°46′–29°30′N	1675	Evergreen Broad-leaved	*Eryngium foetidum* L.<br>*Taraxacum mongolicum* Hand.-Mazz.<br>*Boehmeria nivea* Gaudich.<br>*Rubus amabilis* Focke<br>*Lonicera japonica* Thunb.<br>*Actinidia callosa* Lindl.<br>*Morus alba* L.<br>*Broussonetia papyrifera* L.<br>*Akebia quinata* Houtt.<br>*Celastrus gemmatus* Loes.
CQ2	2023/7/10			1740	Evergreen Broad-leaved	
CQ3	2023/7/10			1763	Evergreen Broad-leaved	
CQ4	2023/7/15			1801	Mixed Coniferous-	
CQ5	2023/7/16			1840	Mixed Coniferous-	
CQ6	2023/7/16			1837	Mixed Coniferous-	
CQ7	2023/7/18			1546	Evergreen Broad-leaved	
CQ8	2023/7/19			1680	Evergreen Broad-leaved	
HN1	2024/7/21	Huping Mountain	110°29′–110°59′E, 29°58′–30°08′N	1022	Evergreen Broad-leaved	*Rubus amabilis* Focke<br>*Actinidia melanandra* Franch.<br>*Dalbergia latifolia* Roxb.<br>*Carpinus turczaninowii* Hance<br>*Akebia quinata* Houtt.<br>*Cornus controversa* Hemsl.<br>*Broussonetia papyrifera* L.
HN2	2024/7/21			1080	Evergreen Broad-leaved	
HN3	2024/7/22			1532	Evergreen Broad-leaved	
HN4	2024/7/22			1618	Evergreen Broad-leaved	
HN5	2024/7/23			1640	Evergreen Broad-leaved	
HN6	2024/7/24			1769	Evergreen Broad-leaved	
HN7	2024/7/24			1821	Evergreen Broad-leaved	
HN8	2024/7/25			1325	Evergreen Broad-leaved	
C1	2024/11/12	*Yongshun Mingfa* Musk Deer Farm	110°58′E, 29°01′N	801	Plantation Forests (Pinus massoniana, Cinnamomum camphora, Fruit Trees)	**Hand-picked fresh foliage**: Mulberry leaves, Paper mulberry leaves, Plum leaves, etc. (During winter months, kiln-dried variants of the above leaves are used from storage)<br><br>**Supplemental Feeds:** Diced Pumpkin, Diced Carrots, Soybean Meal, Cornmeal, etc.
C2						
C3						
C4						
C5						
C6						
C7						
C8						

(**Note:** Geographic coordinates of wild groups are withheld due to confidentiality agreements and endangered species protection protocols.)

Table S2. Statistical results of raw sequencing data processing

Sample ID	Raw_Base (G)	Clean_Base (G)	Clean_Q20 (%)	Clean_Q30 (%)	Clean_GC (%)	Effective (%)
C6	7.38	7.11	97.87	94.89	50.52	96.34
CQ1	8.34	8.23	98.65	96.03	61.57	98.61
CQ3	9.39	9.3	98.88	96.67	50.26	99.04
CQ4	7.85	7.78	98.53	95.77	62.22	99.1
CQ5	8.45	8.27	98.95	96.85	46.09	97.89
CQ6	7.78	7.68	98.82	96.46	52.36	98.8
CQ7	7.35	7.27	98.72	96.23	58.68	98.92
HN1	9.02	8.92	98.78	96.29	60.89	98.95
HN2	7.55	7.44	98.66	96.06	61.14	98.42
HN3	8.45	8.37	98.57	95.86	62.28	98.97
HN5	9.08	8.9	98.71	96.24	51.97	98.08
HN6	9.05	8.97	98.81	96.41	60.32	99.2
HN7	8.09	8.01	98.45	95.48	63.04	99.06
HN8	7.58	7.52	98.61	95.8	62.76	99.21
C1	7.16	7.11	98.97	96.91	46.71	99.27
C2	8.85	8.65	98.9	96.82	48.48	97.7
C3	8.84	8.73	99.04	97.03	48.79	98.79
C4	7.52	7.46	98.87	96.48	51.67	99.12
C5	7.24	7.18	98.99	96.86	47.61	99.09
C7	8.85	8.77	99.03	96.99	49.66	99.06
C8	9.81	9.69	98.71	96.16	60.36	98.83
CQ2	8.97	8.88	98.55	95.84	47.68	98.97
CQ8	8	7.94	98	94.46	58.61	99.21
HN4	10.43	10.31	97.51	93.4	63.26	98.78

Table S3. Statistical results of Alpha diversity analysis at the genus level

Sample ID	ACE	chao1	shannon	simpson	observed_species	goods_coverage
CQ1	3663.47489932896	3653.64705882353	5.01307398935823	0.982092208105953	3638	1
CQ2	2992.49701252622	3011	2.03149815966474	0.599619557162896	2868	1
CQ3	3487.43830813451	3513.02631578947	4.34439325341558	0.956428793471086	3437	1
CQ4	3629.76858469776	3633.53125	4.31259504881545	0.956719475154387	3583	1
CQ5	3259.02028674592	3272.96774193548	2.9646761730739	0.762066498824301	3200	1
CQ6	1719.20515020443	1734.1	4.46051990363914	0.972208415921073	1711	1
CQ7	2864.46620617656	2893	3.90487638461777	0.953425804276386	2791	1
CQ8	2900.66123870251	2919.1320754717	3.66727211260372	0.937196642199293	2822	1
HN1	3702.12856412461	3732.28440366972	4.16320854756786	0.953492150511564	3627	1
HN2	3725.69015119089	3720.88679245283	5.28520412082961	0.982721154519007	3709	1
HN3	3443.59882659387	3468.8	3.49916148610855	0.890074561441638	3375	1
HN4	3514.13241062215	3504.9603960396	5.16908048349935	0.986274322812511	3485	1
HN5	3319.66332087019	3308.52142857143	4.38332901091323	0.955161701293851	3271	1
HN6	3530.11812048834	3575.8407079646	3.86428609173757	0.942054820385194	3438	1
HN7	3082.3691102866	3081.16279069767	5.23177120727099	0.986892916163837	3073	1
HN8	3528.8599909126	3519.47524752475	4.89533478094571	0.981588906124958	3492	1
C1	2906.15904429723	2907	4.64246173856765	0.966438475502588	2854	1
C2	2799.44019082443	2842.13432835821	4.45678867723528	0.956785969645451	2746	1
C3	2707.40528813941	2736.69230769231	4.74936212747522	0.973545418674009	2668	1
C4	2993.78758145473	2992.83582089552	3.88542387163902	0.914015229306177	2916	1
C5	2742.51469398416	2749.2358490566	4.21126031717149	0.941917375065333	2683	1
C6	2221.21032811567	2217.51694915254	2.25118958262598	0.699692171839859	2142	1
C7	2647.91227973667	2701.85087719298	3.10368140874199	0.751657891287826	2522	1
C8	3378.32781445371	3373.5625	3.86568042890449	0.862030368557066	3355	1
